# Both Epimutations and Chromosome Aberrations Affect Multiple Imprinted Loci in Aggressive Wilms Tumors

**DOI:** 10.3390/cancers12113411

**Published:** 2020-11-18

**Authors:** Laura Pignata, Orazio Palumbo, Flavia Cerrato, Basilia Acurzio, Enrique de Álava, Josep Roma, Soledad Gallego, Jaume Mora, Massimo Carella, Andrea Riccio, Gaetano Verde

**Affiliations:** 1 Department of Environmental Biological and Pharmaceutical Sciences and Technologies, University of Campania ‘Luigi Vanvitelli’, 81100 Caserta, Italy; laura.pignata@unicampania.it (L.P.); flavia.cerrato@unicampania.it (F.C.); 2 Institute of Genetics and Biophysics ‘Adriano Buzzati-Traverso’ CNR, 80131-Napoli, Italy; basilia.acurzio@igb.cnr.it; 3 Division of Medical Genetics, Fondazione IRCCS “Casa Sollievo della Sofferenza”, 71013 San Giovanni Rotondo (FG), Italy; o.palumbo@operapadrepio.it (O.P.); m.carella@operapadrepio.it (M.C.); 4 Department of Pathology, Institute of Biomedicine of Sevilla (IBiS), Virgen del Rocio University Hospital/CSIC/University of Sevilla/CIBERONC, 41013 Seville, Spain; enrique.alava.sspa@juntadeandalucia.es; 5 Department of Normal and Pathological Cytology and Histology, School of Medicine, University of Seville, 08035 Seville, Spain; 6 Group of Translational Research in Child and Adolescent Cancer, Vall d’Hebron Research Institute-Universitat Autònoma de Barcelona, 08193 Barcelona, Spain; josep.roma@vhir.org (J.R.); sgallego@vhebron.net (S.G.); 7 Pediatric Cancer Center Barcelona (PCCB), Hospital Sant Joan de Déu, Esplugues de Llobregat, 08950 Barcelona, Spain; jmora@sjdhospitalbarcelona.org

**Keywords:** nephroblastoma, genomic imprinting, DNA methylation, chromosome aberrations

## Abstract

**Simple Summary:**

About 7% of all children’s malignancies are represented by the embryonal renal cancer Wilms tumor (WT). Since methylation imprinting alterations at multiple loci dictated by chromosome copy-number variations have been recently demonstrated in adult cancers, we investigated the presence of similar alterations in pediatric malignancies. Our results demonstrated that 35% of WT cases were affected by methylation abnormalities of multiple imprinted loci. However, differently from adult cancers, they were associated with either chromosome aberrations or normal chromosome profiles. Epigenotype–phenotype correlations indicated that these epimutations were more frequent in highly aggressive tumors, suggesting the use of multiple methylation imprinting defects as a new informative marker for WT.

**Abstract:**

The embryonal renal cancer Wilms tumor (WT) accounts for 7% of all children’s malignancies. Its most frequent molecular defect is represented by DNA methylation abnormalities at the imprinted 11p15.5 region. Multiple imprinted methylation alterations dictated by chromosome copy-number variations have been recently demonstrated in adult cancers, raising the question of whether multiple imprinted loci were also affected in WT. To address this issue, we analyzed DNA methylation and chromosome profiles of 7 imprinted loci in 48 WT samples. The results demonstrated that methylation abnormalities of multiple imprinted loci occurred in 35% of the cases, but that they were associated with either chromosome aberrations or normal chromosome profiles. Multiple imprinted methylation changes were correlated with tumor stage and presence of metastasis, indicating that these epimutations were more frequent in highly aggressive tumors. When chromosome profiles were affected, these alterations were extended to flanking cancer driver genes. Overall, this study demonstrates the presence of multiple imprinted methylation defects in aggressive WTs and suggests that the mechanism by which they arise in embryonal and adult cancers is different.

## 1. Introduction

Wilms tumor (WT) is a pediatric renal cancer, which typically affects 1 child per 10,000 worldwide before the age of 15 years. This malignancy is generally sporadic, with only 1–2% of familial cases [1]. Several congenital disorders predispose to WT, including the WAGR syndrome (Wilms tumor, aniridia, genitourinary anomalies, and intellectual disability), and the overgrowth-associated Beckwith-Wiedemann syndrome [1,2]. In addition, recent comprehensive genomic analyses of large cohorts of patients have identified somatic mutations in about forty genes in WTs [1,2,3], but the majority of these genetic variants affect only a very small subset of patients [1,2,3]. Conversely, DNA methylation abnormalities of chromosome 11p15.5 are very common in WTs [4,5,6,7]. This region contains a cluster of genes controlled by genomic imprinting, a mechanism causing a gene to be expressed only from its maternal or its paternal allele. Imprinted genes are generally organized in clusters in the human genome, and each cluster contains a cis-acting element controlling the allele-specific expression of the neighboring genes (namely, imprinting control region, or ICR). The ICRs include a differentially methylated region (DMR) between the two parental chromosomes [8]. The imprinted gene cluster located at 11p15.5 is functionally divided into two domains containing an ICR each, namely the *H19/IGF2:IG-DMR* and *KCNQ10T1:TSS-DMR*. The telomeric domain includes the paternally expressed *Insulin Like Growth Factor 2 *(*IGF2*) gene and the maternally expressed long non-coding RNA *H1*9 gene, and is controlled by the paternally methylated *H19/IGF2:IG-DMR* that is located between these two genes [9]. In a subset of WTs, gain of methylation of the maternal *H19/IGF2:IG-DMR* allele is responsible for biallelic activation of *IGF2* and biallelic silencing of *H19*, thus increasing the activity of a growth-promoting gene and decreasing that of a growth-inhibitory gene [4,5,6,10]. The centromeric domain of the cluster contains several imprinted genes with maternal-specific expression including the growth inhibitor *CDKN1C* and a long non-coding RNA gene (*KCNQ1OT1*) that is normally expressed only from the paternal chromosome. The imprinting of these genes is controlled by the maternally methylated *KCNQ10T1:TSS-DMR*, which overlaps the promoter of *KCNQ1OT1* [9]. In a small subgroup of WTs, loss of DNA methylation of the maternal *KCNQ10T1:TSS-DMR* allele is associated with silencing of *CDKN1C* [11]. Finally, in roughly 40% of WT cases, the maternal 11p15.5 region is lost and the paternal counterpart is duplicated in the tumor cells, a mechanism known as uniparental disomy (UPD); as a consequence, the tumor DNA shows both gain of *H19/IGF2:IG-DMR* methylation and loss of *KCNQ10T1:TSS-DMR* methylation [1].

Contradictory results have been reported concerning the presence of imprinting defects in loci other than the 11p15.5 gene cluster in WTs. A first study performed with microarray on 38 imprinted loci did not identify epigenetic defects outside the 11p15.5 region in WTs [12]. Further studies have described rare methylation defects at GTL2-DLK1 or NNAT/BLCAP loci [13,14]. More recently, expression deregulation of several imprinted genes was reported in this malignancy, although neither DNA methylation nor allele-specific expression of these genes, apart from those of the 11p15.5 locus, was investigated [15]. Therefore, further studies are required to clarify whether and how imprinting defects affect only the 11p15.5 locus or multiple loci in WT.

Methylation aberrations of multiple imprinted loci that are mostly associated with chromosome copy-number variations (CNVs) have been demonstrated in adult lung, breast, colon and liver cancers, demonstrating that in these cases, the epigenetic defects are mostly secondary to genomic changes [16,17]. However, to date, no such study has been performed in WT. To decipher the mechanisms underlying imprinting defects in this malignancy, this study analyzes DNA methylation and chromosome profile in a large cohort of WT cases. We report for the first time that the most aggressive WTs show frequent abnormalities of methylation imprinting at multiple loci. Interestingly, the methylation imprinting defects are associated with either chromosome aberrations or normal chromosome profiles. Taken together, these data indicate multiple imprinting abnormalities as a new informative marker for WT and suggest that the mechanism by which imprinting defects arise in embryonal and adult cancers is different.

## 2. Results

### 2.1. DNA Methylation Defects Affect Multiple Imprinted Loci in WTs

**Figure 1:**
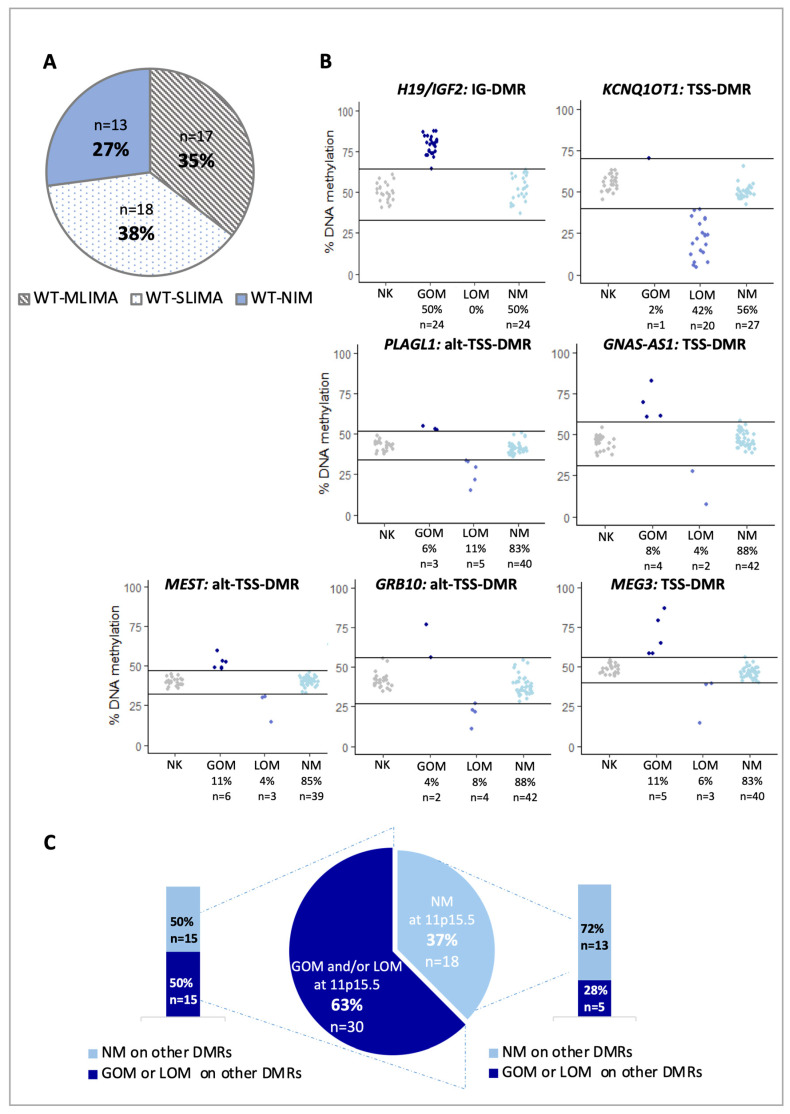
DNA methylation aberrations at imprinted loci in WTs. (**A**) Pie graph showing the number and percentage of the tumors with multi-locus imprinted methylation aberration (WT-MLIMA), the cases with single-locus imprinted methylation aberration (WT-SLIMA) and the cases with normal imprinted methylation (WT-NIM). (**B**) DNA methylation quantification at the indicated imprinted DMRs. The average methylation of the CpGs of each DMR in normal kidney (NK) and WTs was used to generate the dots graphs. Based on the DNA methylation profiles, WTs were stratified in three groups: patients with gain of methylation (GOM), loss of methylation (LOM), or normal methylation (NM). We define WTs with GOM or LOM when the average methylation value of the patients is outside (±) three times the standard deviation of the mean of all normal kidney samples. The horizontal lines indicate the interval of values within three standard deviations of the mean of the normal kidney samples. (**C**) Pie graph showing the percentage of WTs with or without methylation defects at the 11p15.5 region, including both *H19/IGF2:IG-DMR* and *KCNQ10T1:TSS-DMR*. The histograms on the left and right of the pie graph show the percentage of WTs with or without methylation defects at the *PLAGL1*, *GNAS*-AS1, *MEST*, *GRB10* and *MEG3* DMRs. GOM: gain of methylation; LOM: loss of methylation. NM: normal methylation.

In order to investigate whether imprinted methylation outside of the 11p15.5 locus (*H19/IGF2:IG-DMR* and *KCNQ10T1:TSS-DMR*) is affected in WT, we analyzed the DNA methylation profile of five further imprinted DMRs (*PLAGL1, GNAS, MEST, GRB10* and *MEG3*) in 48 WTs and 23 normal kidney tissues, by bisulfite conversion and pyrosequencing. The analyzed DMRs were chosen among the ones that are most frequently affected in imprinting disorders with multi-locus imprinting abnormalities [9,18,19]. Clinical details of the cohort are listed in Table S1. The results demonstrated that methylation imprinting defects were present at more than one imprinted region in 35% of tumors(Wilms tumor with multi-locus imprinted methylation aberration, WT-MLIMA), while 38% of the cases showed gain and/or loss of methylation only at a single locus (Wilms tumor with single-locus imprinted methylation aberration, WT-SLIMA,), and only 27% showed a methylation profile similar to normal kidneys at all tested imprinted regions (Wilms tumor with normal imprinted methylation, WT-NIM,) (Figure 1A). Notably, we confirmed that the methylation defects more frequently affect the 11p15.5 DMRs (50% and 44% for *H19/IGF2:IG-DMR* and *KCNQ10T1:TSS-DMR*, respectively), but also demonstrated significant methylation changes (either loss or gain) at the other DMRs in the tumor tissue with respect to normal kidney, although in a lower number of cases (Figure 1B and Table S2). Moreover, while hypermethylation was consistently found at the *H19/IGF2:IG-DMR*, and hypomethylation was almost always found at the *KCNQ10T1:TSS-DMR*, either loss or gain of methylation was detected at the other DMRs (Figure 1B). These results were further confirmed through the analysis of methylation of a second region of the DMRs by a different technique, the methylation-specific multiplex ligation-dependent probe amplification (MS-MLPA, Table S3). In addition, we found that the methylation alterations at the 11p15.5 DMRs were generally greater than the other epimutations (Figure S1 and Table S2). Furthermore, when multiple DMRs were affected, these more frequently included the *H19/IGF2:IG-DMR* and the *KCNQ10T1:TSS-DMR* (Figure 1C).

### 2.2. Epimutations at Multiple Imprinted Loci are Associated with Tumor Aggressiveness in WT

**Figure 2:**
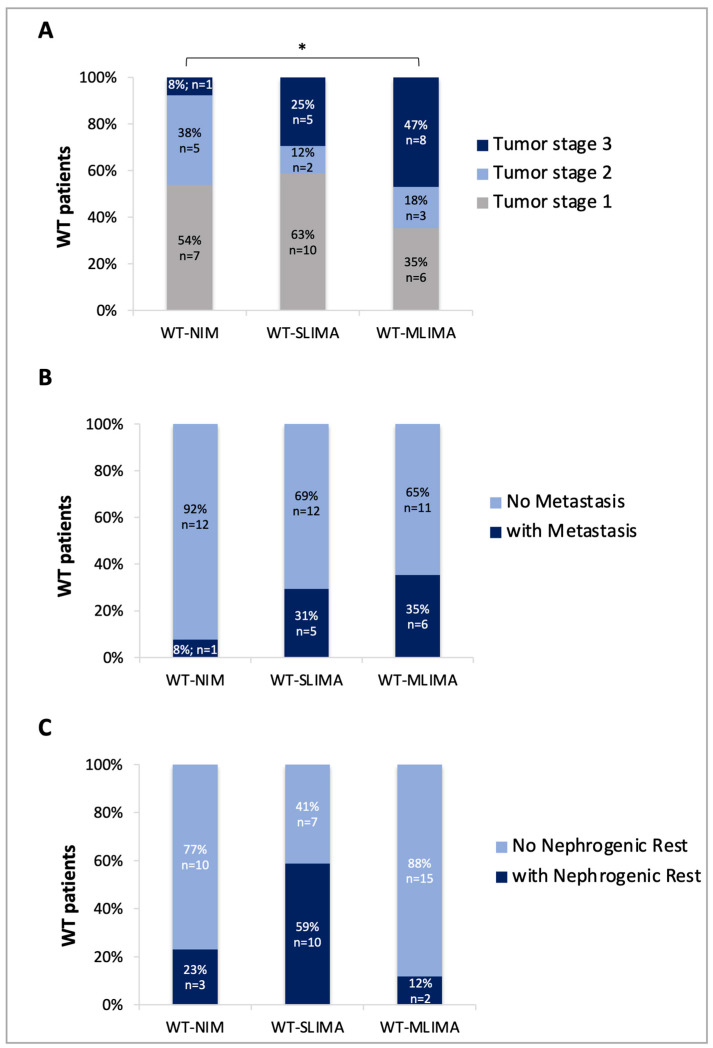
Correlation between epigenotype and phenotype. Distribution of the tumor stages (**A**), metastasis (**B**), or nephrogenic rest (**C**) for 47 patients of which clinical information was available. WT-MLIMA, patients with multilocus imprinted methylation aberrations; WT-SLIMA, patients with single-locus imprinted methylation aberrations; WT-NIM, patients with normal imprinted methylation. * *p* less than or equal to 0.05, chi-square test.

To investigate if the observed tumor epigenotypes had any clinical relevance, we correlated them with the available histopathological features. We found that the WT-MLIMA cases generally corresponded to tumors with more advanced stage and were associated with more frequent metastases compared with the WT-SLIMA and WT-NIM groups, indicating that the multiple imprinted methylation defects are mainly associated with the most aggressive tumors (Figure 2A,B). Conversely, the presence of nephrogenic rests (precancerous lesions) was more frequently associated with the WTs showing methylation changes only at 11p15.5 (WT-SLIMA, Figure 2C).

### 2.3. The Epimutations at Imprinted Loci are Associated with Different Chromosome Profiles

**Figure 3:**
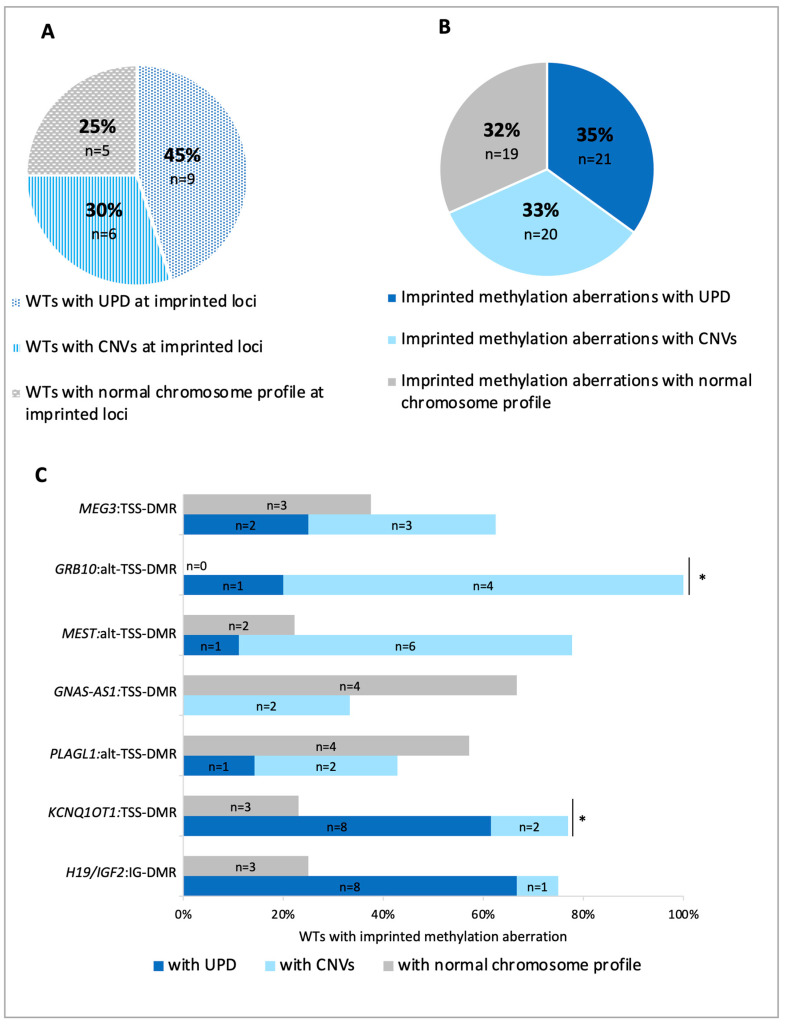
Chromosome status of imprinted loci. (**A**) Distribution of WTs with or without chromosome aberrations (CNVs or UPD) in at least one imprinted differentially methylated region (DMR )analyzed. (**B**) Distribution of methylation imprinting defects associated with CNVs, UPD or normal chromosome profiles. (**C**) Correlation between DNA methylation defects and chromosome profile at each imprinted locus. * *p* less than or equal to 0.05; binomial test.

To investigate whether chromosome alterations underly methylation imprinting defects as in adult cancers [16,17], we analyzed the genomic profiles of 20 WTs with methylation changes by CytoScan high-definition array. The results showed that chromosome aberrations, including either CNVs or UPD, were present at least in one imprinted locus in the majority of WTs (Figure 3A and Table S4). CNVs and UPD overlapped 33% and 35% respectively, of the DMRs with methylation changes (Figure 3B). The remaining 32% of the abnormally methylated DMRs showed normal chromosome profiles at these loci (Figure 3B). Both CNVs and UPD more frequently affected the DMRs of *H19*, *KCNQ1OT1*, *MEST*, *GRB10* and *MEG3,* while the methylation changes of *GNAS* and *PLAGL1* DMRs were mostly associated with normal chromosome profiles (Figure 3C).

**Figure 4:**
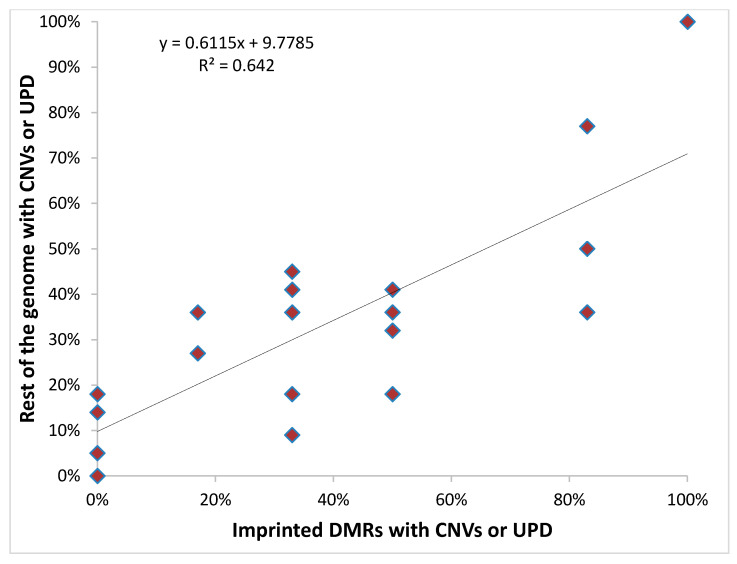
Correlation between chromosome aberrations of imprinted DMRs and the rest of the genome in WTs with methylation imprinting defects.

The results of the single nucleotide polymorphism-array (SNP-array) analysis showed that CNVs and UPD were generally not limited to the imprinted DMRs but also affected non-imprinted genomic regions and, interestingly, patients with normal chromosome profiles at imprinted loci generally also had fewer chromosome alterations in the rest of the genome compared to the other cases (Figure 4, Figure S2). Remarkably, WT-2 shows UPD, duplication or amplification of almost the entire genome (Figure S2, WT-2).

### 2.4. Chromosome Alterations at Imprinted Loci Include Flanking Cancer Driver Genes

**Figure 5:**
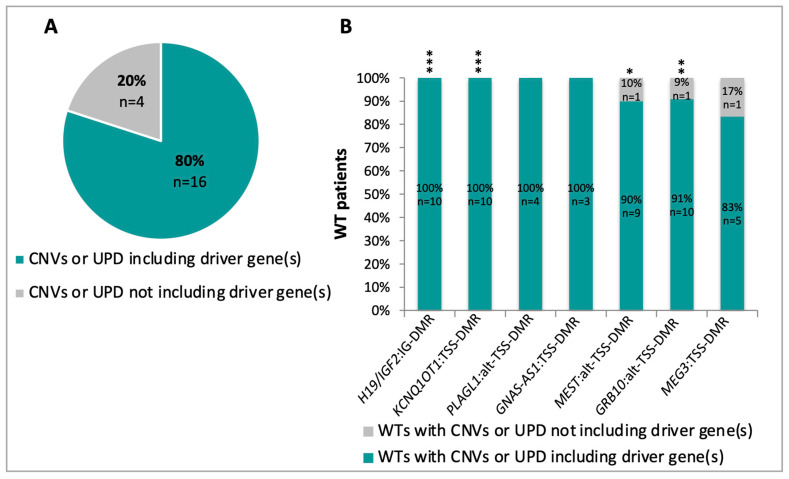
(**A**) Distribution of WTs with CNVs or UPD at all imprinted DMRs including or not including flanking cancer driver genes. (**B**) Percentage of WTs with CNVs or UPD at indicated imprinted DMRs including or not including flanking cancer driver genes. * *p* less than or equal to 0.05, ** *p* less than or equal to 0.01, *** *p* less than or equal to 0.001, binomial test.

In two thirds of the abnormally methylated imprinted DMRs, the methylation defects were dictated by chromosome events that generally affected regions larger than the imprinted loci. We investigated if these chromosome alterations also included previously identified cancer driver genes [1,3]. We found that this was true in 80% of our cases (Figure 5A, Table 1). Indeed, the chromosome alterations affecting the *H19*, *KCNQ1OT1*, *PLAGL1* and *GNAS* DMRs also affected one or more WT driver genes in all analyzed cases, and the chromosome variants affecting the *MEST*, *GRB10* and *MEG3* DMRs also affected tumor driver genes in at least 83% of cases (Figure 5B, Table 1).

## 3. Discussion

Alterations of DNA methylation of the 11p15.5 imprinted gene cluster are the most frequent molecular defect found in WT so far [4,5,6]. Whether these epimutations are limited to this genomic region or affect other imprinted loci is still undefined. In this work, we demonstrated that a large subgroup of WTs showed methylation imprinting abnormalities at multiple loci, including *H19*, *KCNQ1OT1*, *PLAGL1*, *GNAS*, *MEST*, *GRB10* and *MEG3*. We observed that the WT cases with epimutations at more than one imprinted chromosome region correspond to more advanced tumor stages and have more frequent metastases with respect to the cases with single-locus defects or normal imprinting, suggesting that multiple imprinting defects arise mostly during the late stages of tumorigenesis. Consistent with these data, it has been recently proposed that 44 genes controlled by DNA methylation, including multiple imprinted genes, can be a signature for metastasis formation in WT [20].

The methylation alterations at *H19/IGF2:IG-DMR* and *KCNQ10T1:TSS-DMR* detected in the tumor samples were generally more intense than those affecting the other imprinted loci. Aberrant methylation of the 11p15.5 DMRs represents the most frequent epimutation found in our tumor samples, including those in stage 1. Moreover, multiple imprinting-associated epimutations affect WTs with 11p15.5 defects more frequently than WTs without 11p15.5 defects. Taken together, these data suggest that the 11p15.5 epimutations occur earlier than those at other imprinted loci during malignant transformation. Accordingly, it has been reported that hypermethylation at *H19/IGF2:IG-DMR* is present in the premalignant clonal expansions of WTs, representing an early event in Wilms tumorigenesis [21,22], and as constitutional epimutation in the Beckwith-Wiedemann syndrome cases with high predisposition to WTs [5,23,24,25,26].

The mechanisms underlying the imprinting epimutations in WT have not been defined yet. Here, we report that a large subgroup of methylation imprinting defects is associated with *cis* chromosome aberrations, including duplication, deletion and UPD. We found that the great majority of CNVs and UPD at imprinted loci cover previously identified WT driver genes [1,3], suggesting that, in these cases, imprinting epimutations are mostly secondary to genomic alterations occurring at the flanking driver gene/s, as previously demonstrated in adult cancers [16,17]. However, in 25% of the WT cases and 32% of the abnormally methylated DMRs, imprinting epimutations are associated with normal chromosome profiles. Lower figures are reported by Martin-Trujillo and collaborators for breast, lung, liver and colon cancers [16]. What is the mechanism driving the epigenetic defect in these cases is still an open question. Since either loss or gain of imprinted methylation are simultaneously present in WTs, it is unlikely that these epimutations are caused by defects in trans-acting factors involved in the somatic maintenance of imprinted methylation. On the other hand, it has been previously reported that hypermethylation at *H19/IGF2:IG-DMR* is rarely linked to microdeletions or microinsertions of the maternal *H19/IGF2:IG-DMR* allele in non-syndromic WTs [4,27]. Given that the SNP-array employed in our study detects only CNVs larger than 1 kb, we cannot exclude that a small subset of imprinting abnormalities in WTs is associated with *cis* genetic alterations not detectable by this method. However, we and others previously demonstrated that a large cohort of WTs with hypermethylated *H19/IGF2:IG-DMR* did not show any genetic mutation of this genomic region [5,23,28]. Accordingly, the association between multiple imprinting epimutations with normal *cis* chromosome profiles demonstrated in this study suggests that methylation imprinting aberrations in WTs arise as a consequence of either defective methylation maintenance or stochastic or environment-driven events.

Bjornsson and colleagues analyzed methylation of 38 imprinted loci in WTs by a microarray-based assay, and in contrast to our finding, they did not find any methylation imprinting alterations outside the 11p15.5 region [12]. The discrepancy between the two studies could be due to the different sensitivity of the methods used, as well as the relatively few CpGs investigated in the microarray of the older study. We believe that the higher sensitivity of the bisulfite conversion and pyrosequencing method used in this study can enable to detect methylation defects that are difficult to identify by microarray. DNA methylation of the *MEG3* locus was investigated by Astuti and collaborators [13] in 40 WTs, and found to be hypermethylated in 2.5% of the samples. We found hypermethylation and hypomethylation of *MEG3* in 11% and 6% respectively, of the 48 WTs investigated. As in the Bjornsson’s study, this discrepancy could be explained with the use of different techniques, and in particular, with the higher number of CpGs we investigated by pyrosequencing with respect to the methylation-specific polymerase chain reaction (MS-PCR) used in the previous study. Hubertus and collaborators [14] investigated the methylation of the *NNAT* locus by pyrosequencing and demonstrated a significant hypomethylation associated with upregulation of *NNAT* and the nearby *BLCAP* gene in the majority of the 45 WTs tested. Although we were unable to analyze *NNAT* methylation in our samples, this study is consistent with our results. Finally, Gadd and collaborators [3] analyzed genome-wide DNA methylation of a large cohort of WT cases by microarray. Consistent with our results, they reported that the only imprinted locus with coordinately differentially methylated CpGs is *H19*. This study does not focus on regions with inconsistent methylation changes, and this may explain why they did not report the finding of both hypo- and hyper-methylation in the other imprinted loci.

The DMRs play a key role in the regulation of the allele-specific expression of the surrounding imprinted genes. DNA methylation aberrations at these regions, indeed, are generally associated with loss of imprinting (either biallelic activation or biallelic silencing) of the surrounding genes [8,9]. The *H19/IGF2:IG-DMR* hypermethylation, for example, is coupled to the loss of imprinting of *IGF2* and *H19* in several congenital diseases and cancers, including WTs [5,22,29]. Several studies reported deregulation of the global expression of several imprinted genes in WTs, although their allele-specific expression was not investigated [15,30]. Due to the non-availability of RNA samples, we have been unable to test gene expression in our samples. Therefore, whether the multiple methylation imprinting defects of WTs identified in our study are associated with the loss of the allele-specific expression of the surrounding genes remains an open question that needs further investigation. It should be considered, however, that the inconsistency of the methylation changes of the imprinted loci outside of 11p15.5 suggests that apart from *H19/IGF2* and *KCNQ1OT1/CDKN1C,* the other imprinted genes do not likely act as drivers of cancer progression in WT.

Due to the rarity of this cancer type, a limitation of this study is the reduced sample size, particularly of the cases tested for the presence of CNVs. Further studies on larger cohorts of WTs are needed to validate the use of multiple methylation imprinting defects as informative markers for tumor progression and patient stratification.

## 4. Materials and Methods

### 4.1. Patients

48 WT and 23 normal kidney (NK) tissues have been recruited from Spanish pediatric oncology units affiliated to ‘Hospital Universitario Virgen del Rocío’ (Sevilla, Spain, 31 WTs and 21 NK), ‘Hospital Vall d’Hebron’ (Barcelona, Spain, 17 WTs) and ‘Hospital San Joan de Deu’ (Barcelona, Spain, 2NK). All tumors were histologically diagnosed as WT. This study was approved by the ethical committees of University of Campania ‘Luigi Vanvitelli’ (Prot. 0010423/i-05/05/2020), Hospital Universitario ‘Virgen del Rocío’ (52a21bdb0fe951adc9ea75883b032a4d40d37033–15/05/2020), Hospital ‘Vall d’Hebron’ (PR(AG)276/2020–11/08/2020) and ‘Hospital San Joan de Deu’ (BB-CCM-004–20/12/2018).

### 4.2. DNA Methylation Analysis

One μg of genomic DNA extracted from tumor tissue was treated with sodium bisulfite by using the EpiTect Bisulfite kit (Qiagen-Italia, Milan, Italy) following the manufacturer’s protocol. About 100 ng of converted DNA were amplified by using the PyroMark PCR kit (Qiagen-Italia, Milan, Italy) in a final volume of 25 μL. Fifteen μL of PCR product was used for quantitative DNA methylation by pyrosequencing on a Pyromark Q48 Autoprep system with the PyroMark Q48 Advanced CpG Reagents Kit (Qiagen-Italia, Milan, Italy) and PyroMark Q48 Magnetic Beads. Results were analyzed by using the Pyromark Q48 Autoprep software. The primers used for PCR amplification and pyrosequencing were designed with Pyromark Assay Design SW 2.0 and are reported in Table S5.

Methylation analysis of several imprinted DMRs was performed on MLIMA samples also by MS-MLPA (SALSA MS-MLPA Kit ME034-B1, MRC-Holland, Amsterdam, The Netherlands) following the manufacturer’s instructions.

### 4.3. Chromosome Microarray Analysis

In order to analyze the chromosome profiles of WTs, high-resolution single nucleotide polymorphism array (SNP-based array) has been performed using the CytoScan HD array (Thermo Fisher Scientific, Waltham, MA, USA), which contains more than 2.6 million markers for CNVs analysis and approximately 750,000 SNP probes able to genotype with an accuracy greater than 99%, following the manufacturer’s instructions. Data analysis was performed using Chromosome Analysis Suite software version 4.0 (Thermo Fisher Scientific, Waltham, MA, USA) following a standardized pipeline. Briefly: (i) the raw data file (.CEL) of each sample enrolled for the study was normalized using the default options, and (ii) an unpaired analysis has been performed using as baseline 270 HapMap samples to obtain chromosome copy-numbers’ value and regions of homozygosity (ROH) from. CEL files. The amplified and/or deleted regions, on the other hand, have been detected using a standard Hidden Markov Model (HMM) method. We have not applied any minimum size threshold for CNVs and ROHs calling and reporting. To identify clinical or functionally relevant genomic variants, all chromosomal alterations have been compared to those collected in an internal database of 4000 patients studied by SNP arrays since 2010, and public databases, including the database of genomic variant (http://projects.tcag.ca/variation/), DECIPHER (https://decipher.sanger.ac.uk/) and ClinVar (https://www.ncbi.nlm.nih.gov/clinvar/). Base pair positions, information about genomic regions and genes affected by CNVs and/or ROHs have been derived from the UCSC Genome Browser using build GRCh37 chromosome assembly. The clinical significance of each rearrangement detected has been assessed following the American College of Medical Genetics guidelines [31].

## 5. Conclusions

We demonstrated the presence of multiple methylation imprinting defects in aggressive WTs, indicating a new informative marker for this malignancy. Additionally, we showed that differently from adult cancers, multiple imprinting epimutations in WTs can be associated with either chromosome aberrations or normal chromosome profiles, suggesting that the mechanism by which imprinting defects arise may differ in embryonal and adult cancers.

## Figures and Tables

**Table 1:** WTs driver genes flanking the indicated imprinted DMRs.

Chromosome	Imprinted DMR	WT Driver Genes
6	*PLAGL1:*alt-TSS-DMR	*XPO5*, *LIN28B*
7	*MEST*:alt-TSS-DMR	*ACTB*, *TNRC18*
7	*GRB10:*alt-TSS-DMR	*ACTB*, *TNRC18*
11	*H19/IGF2:*IG-DMR	*IGF2*, *WT1*
11	*KCNQ1OT1:*TSS-DMR	*IGF2*, *WT1*
14	*MEG3*:TSS-DMR	*SIX1*, *MAX*, *DICER1*
20	*GNAS-AS1:*TSS-DMR	*ASXL1*

Italics: Genes.

## Supplementary Materials

The following are available online at https://www.mdpi.com/2072-6694/12/11/3411/s1, Figure S1: Average of DNA methylation aberrations at imprinted loci in WTs. Figure S2: Karyoview of WTs analyzed by high-resolution SNP-Arrays. Table S1: Clinical features of each tumor analyzed in the present study. Table S2: Methylation values of single CpGs obtained by pyrosequencing at eight imprinting DMRs. Table S3: Comparison between MS-MLPA and pyrosequencing methylation data of seven DMRs in MLIMA samples. Table S4: DNA methylation and chromosome profile of WTs at indicated imprinted DMRs. Table S5: Primers used for pyrosequencing analysis.

## References

[1] Treger T.D., Chowdhury T., Pritchard-Jones K., Behjati S. (2019). The genetic changes of Wilms tumour. Nat. Rev. Nephrol..

[2] Brioude F., Kalish J.M., Mussa A., Foster A.C., Bliek J., Ferrero G.B., Boonen S.E., Cole T., Baker R., Bertoletti M. (2018). Expert consensus document: Clinical and molecular diagnosis, screening and management of Beckwith-Wiedemann syndrome: An international consensus statement. Nat. Rev. Endocrinol..

[3] Gadd S., Huff V., Walz A.L., Ooms A.H.A.G., Armstrong A.E., Gerhard D.S., Smith M.A., Auvil J.M.G., Meerzaman D., Chen Q.R. (2017). A Children’s Oncology Group and TARGET initiative exploring the genetic landscape of Wilms tumor. Nat. Genet..

[4] Scott R.H., Douglas J., Baskcomb L., Huxter N., Barker K., Hanks S., Craft A., Gerrard M., Kohler J.A., Levitt G.A. (2008). Constitutional 11p15 abnormalities, including heritable imprinting center mutations, cause nonsyndromic Wilms tumor. Nat. Genet..

[5] Cerrato F., Sparago A., Verde G., De Crescenzo A., Citro V., Cubellis M.V., Rinaldi M.M., Boccuto L., Neri G., Magnani C. (2008). Different mechanisms cause imprinting defects at the IGF2/H19 locus in Beckwith-Wiedemann syndrome and Wilms’ tumour. Hum. Mol. Genet..

[6] Anvar Z., Acurzio B., Roma J., Cerrato F., Verde G. (2019). Origins of DNA methylation defects in Wilms tumors. Cancer Lett..

[7] Murrell A., Ito Y., Verde G., Huddleston J., Woodfine K., Silengo M.C., Spreafico F., Perotti D., De Crescenzo A., Sparago A. (2008). Distinct methylation changes at the IGF2-H19 locus in congenital growth disorders and cancer. PLoS ONE.

[8] Ferguson-Smith A.C., Bourc’his D. (2018). The discovery and importance of genomic imprinting. Elife.

[9] Monk D., Mackay D.J.G., Eggermann T., Maher E.R., Riccio A. (2019). Genomic imprinting disorders: Lessons on how genome, epigenome and environment interact. Nat. Rev. Genet..

[10] Yoshimizu T., Miroglio A., Ripoche M.A., Gabory A., Vernucci M., Riccio A., Colnot S., Godard C., Terris B., Jammes H. (2008). The H19 locus acts in vivo as a tumor suppressor. Proc. Natl. Acad. Sci. USA.

[11] Brzezinski J., Shuman C., Choufani S., Ray P., Stavropoulos D.J., Basran R., Steele L., Parkinson N., Grant R., Thorner P. (2017). Wilms tumour in Beckwith-Wiedemann Syndrome and loss of methylation at imprinting centre 2: Revisiting tumour surveillance guidelines. Eur. J. Hum. Genet..

[12] Bjornsson H.T., Brown L.J., Fallin M.D., Rongione M.A., Bibikova M., Wickham E., Fan J.B., Feinberg A.P. (2007). Epigenetic specificity of loss of imprinting of the IGF2 gene in Wilms tumors. J. Natl. Cancer Inst..

[13] Astuti D., Latif F., Wagner K., Gentle D., Cooper W.N., Catchpoole D., Grundy R., Ferguson-Smith A.C., Maher E.R. (2005). Epigenetic alteration at the DLK1-GTL2 imprinted domain in human neoplasia: Analysis of neuroblastoma, phaeochromocytoma and Wilms’ tumour. Br. J. Cancer.

[14] Hubertus J., Zitzmann F., Trippel F., Müller-Höcker J., Stehr M., von Schweinitz D., Kappler R. (2013). Selective methylation of CpGs at regulatory binding sites controls NNAT expression in Wilms tumors. PLoS ONE.

[15] Hubertus J., Lacher M., Rottenkolber M., Müller-Höcker J., Berger M., Stehr M., von Schweinitz D., Kappler R. (2011). Altered expression of imprinted genes in Wilms tumors. Oncol. Rep..

[16] Martin-Trujillo A., Vidal E., Monteagudo-Sánchez A., Sanchez-Delgado M., Moran S., Hernandez Mora J.R., Heyn H., Guitart M., Esteller M., Monk D. (2017). Copy number rather than epigenetic alterations are the major dictator of imprinted methylation in tumors. Nat. Commun..

[17] Goovaerts T., Steyaert S., Vandenbussche C.A., Galle J., Thas O., Van Criekinge W., De Meyer T. (2018). A comprehensive overview of genomic imprinting in breast and its deregulation in cancer. Nat. Commun..

[18] Sanchez-Delgado M., Riccio A., Eggermann T., Maher E.R., Lapunzina P., Mackay D., Monk D. (2016). Causes and Consequences of Multi-Locus Imprinting Disturbances in Humans. Trends Genet..

[19] Cubellis M.V., Pignata L., Verma A., Sparago A., Del Prete R., Monticelli M., Calzari L., Antona V., Melis D., Tenconi R. (2020). Loss-of-function maternal-effect mutations of PADI6 are associated with familial and sporadic Beckwith-Wiedemann syndrome with multi-locus imprinting disturbance. Clin. Epigenet..

[20] Guerra J.V.D.S., Pereira B.M.S., Cruz J.G.V.D., Scherer N.M., Furtado C., Montalvão de Azevedo R., Oliveira P.S.L., Faria P., Boroni M., de Camargo B. (2019). Genes Controlled by DNA Methylation Are Involved in Wilms Tumor Progression. Cells.

[21] Coorens T.H.H., Treger T.D., Al-Saadi R., Moore L., Tran M.G.B., Mitchell T.J., Tugnait S., Thevanesan C., Young M.D., Oliver T.R.W. (2019). Embryonal precursors of Wilms tumor. Science.

[22] Okamoto K., Morison I.M., Taniguchi T., Reeve A.E. (1997). Epigenetic changes at the insulin-like growth factor II/H19 locus in developing kidney is an early event in Wilms tumorigenesis. Proc. Natl. Acad. Sci. USA.

[23] Cui H., Niemitz E.L., Ravenel J.D., Onyango P., Brandenburg S.A., Lobanenkov V.V., Feinberg A.P. (2001). Loss of imprinting of insulin-like growth factor-II in Wilms’ tumor commonly involves altered methylation but not mutations of CTCF or its binding site. Cancer Res..

[24] Mussa A., Peruzzi L., Chiesa N., De Crescenzo A., Russo S., Melis D., Tarani L., Baldassarre G., Larizza L., Riccio A. (2012). Nephrological findings and genotype-phenotype correlation in Beckwith-Wiedemann syndrome. Pediatr. Nephrol..

[25] Mussa A., Russo S., De Crescenzo A., Freschi A., Calzari L., Maitz S., Macchiaiolo M., Molinatto C., Baldassarre G., Mariani M. (2016). (Epi)genotype-phenotype correlations in Beckwith-Wiedemann syndrome. Eur. J. Hum. Genet..

[26] Mussa A., Molinatto C., Baldassarre G., Riberi E., Russo S., Larizza L., Riccio A., Ferrero G.B. (2016). Cancer Risk in Beckwith-Wiedemann Syndrome: A Systematic Review and Meta-Analysis Outlining a Novel (Epi)Genotype Specific Histotype Targeted Screening Protocol. J. Pediatr..

[27] Riccio A. (2008). Wilms tumor and constitutional epigenetic defects. Nat. Genet..

[28] Riccio A., Sparago A., Verde G., De Crescenzo A., Citro V., Cubellis M.V., Ferrero G.B., Silengo M.C., Russo S., Larizza L. (2009). Inherited and Sporadic Epimutations at the IGF2-H19 locus in Beckwith-Wiedemann syndrome and Wilms’ tumor. Endocr. Dev..

[29] Sparago A., Russo S., Cerrato F., Ferraiuolo S., Castorina P., Selicorni A., Schwienbacher C., Negrini M., Ferrero G.B., Silengo M.C. (2007). Mechanisms causing imprinting defects in familial Beckwith-Wiedemann syndrome with Wilms’ tumour. Hum. Mol. Genet..

[30] Dekel B., Metsuyanim S., Schmidt-Ott K.M., Fridman E., Jacob-Hirsch J., Simon A., Pinthus J., Mor Y., Barasch J., Amariglio N. (2006). Multiple imprinted and stemness genes provide a link between normal and tumor progenitor cells of the developing human kidney. Cancer Res..

[31] Kearney H.M., Thorland E.C., Brown K.K., Quintero-Rivera F., South S.T., A Working Group of the American College of Medical Genetics (ACMG) Laboratory Quality Assurance Committee (2011). American College of Medical Genetics standards and guidelines for interpretation and reporting of postnatal constitutional copy number variants. Genet. Med..

