# Supplementary material for: Both Epimutations and Chromosome Aberrations Affect Multiple Imprinted Loci in Aggressive Wilms Tumors
Source: Cancers (Basel). 2020 Nov 18;12(11):3411. doi: 10.3390/cancers12113411 (PMC7698742; doi:10.3390/cancers12113411)

Supplementary Materials

Both Epimutations and Chromosome Aberrations Affect Multiple Imprinted Loci in Aggressive Wilms Tumors

Laura Pignata, Orazio Palumbo, Flavia Cerrato, Basilia Acurzio, Enrique de Álava, Josep Roma, Soledad Gallego, Jaume Mora, Massimo Carella, Andrea Riccio and Gaetano Verde


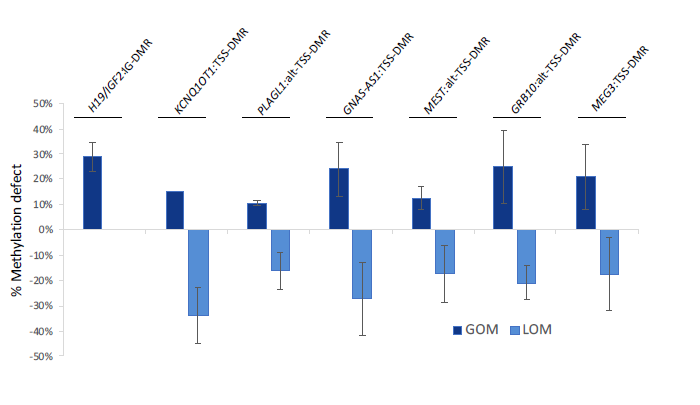


Figure S1. Average level of gain of methylation (GOM) or loss of methylation (LOM) for each DMR normalized against the average of methylation of normal kidneys.


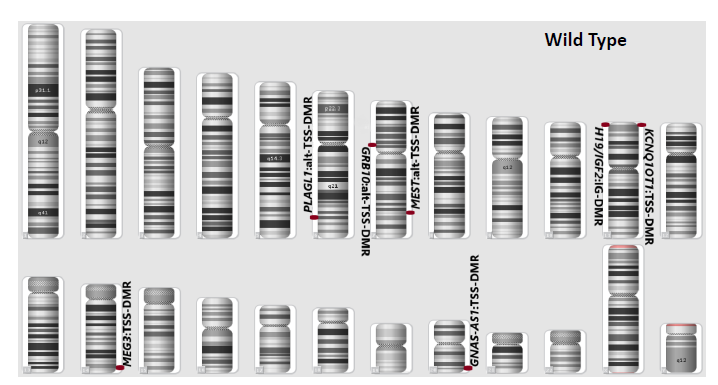


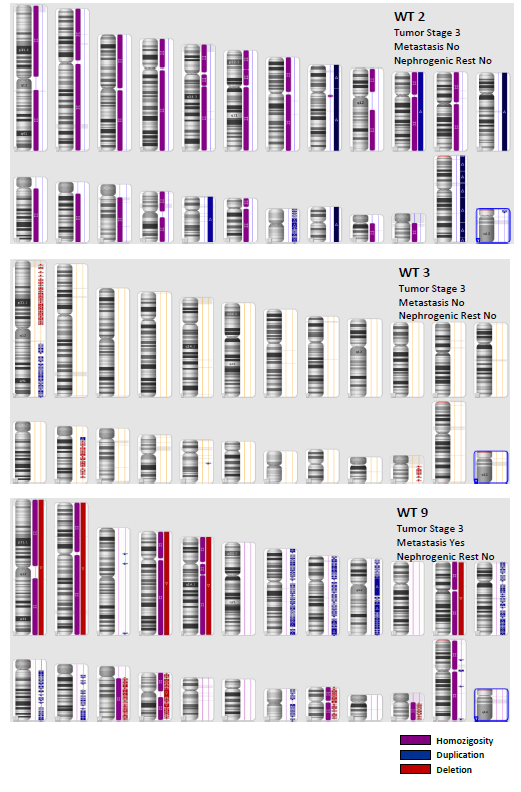


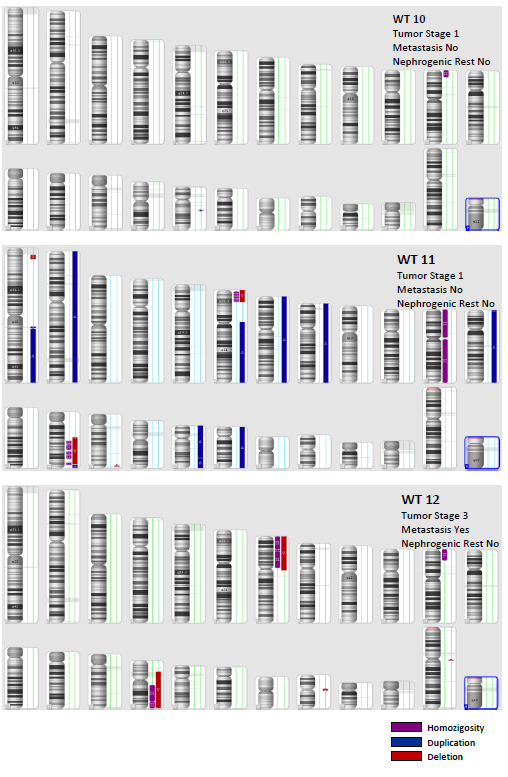


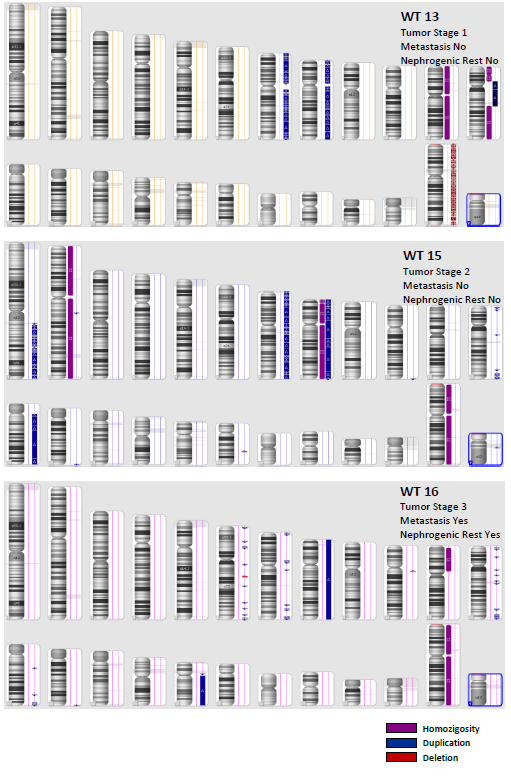


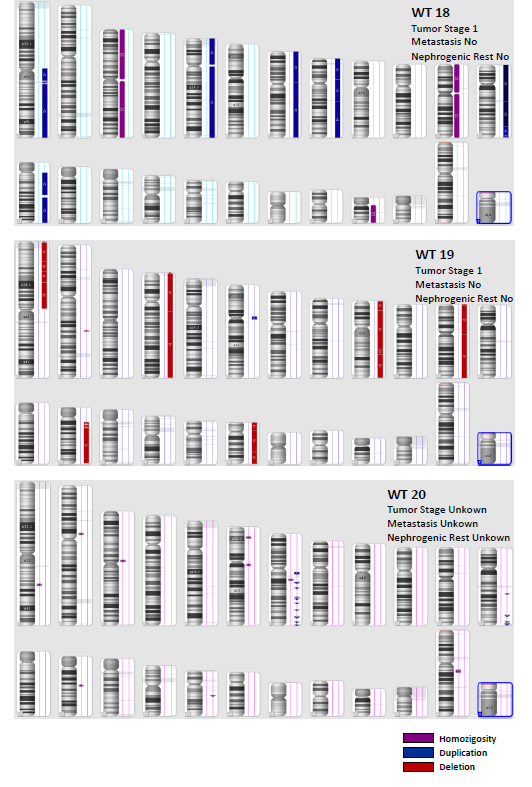


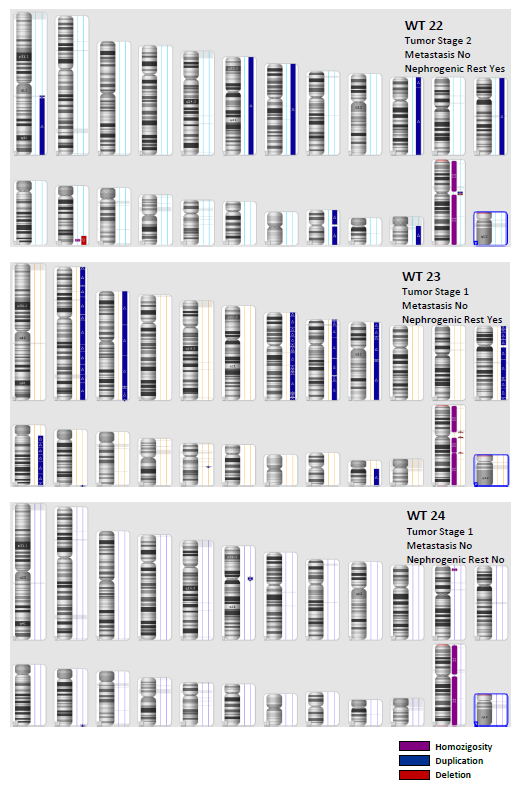


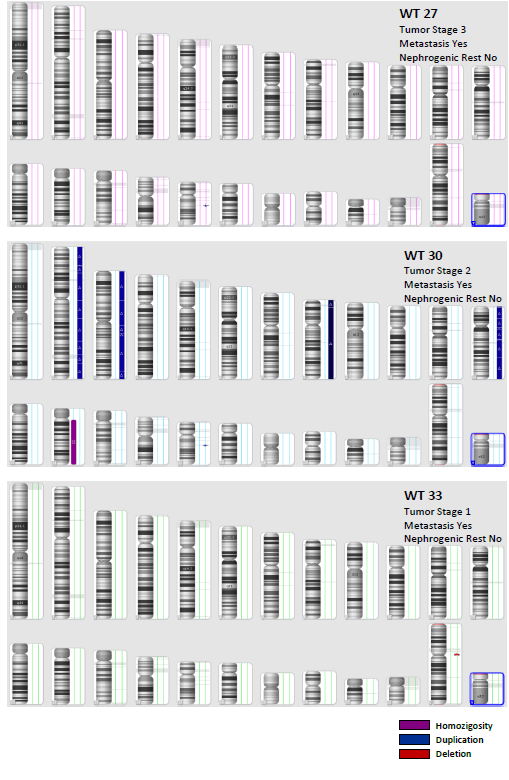


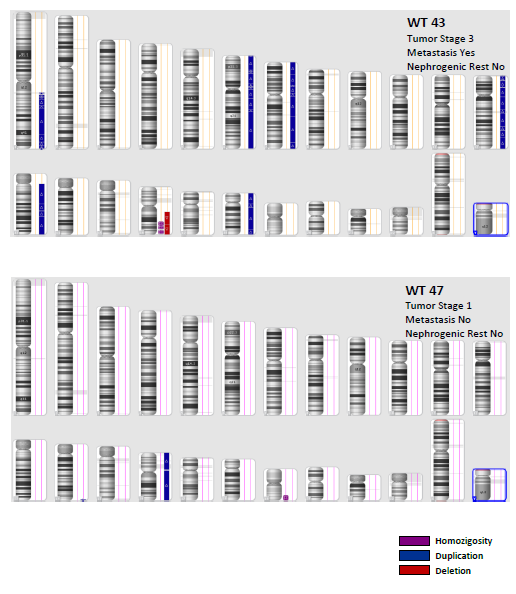


Figure S2. Karyoview of WTs analyzed by high-resolution SNP-Arrays. Regions of homozygosity are indicated by purple bars, while genomic duplications and deletions are indicated by blue bars and red bars, respectively.

Table S3. Comparison between MS-MLPA and pyrosequencing methylation data of seven DMRs in MLIMA samples.


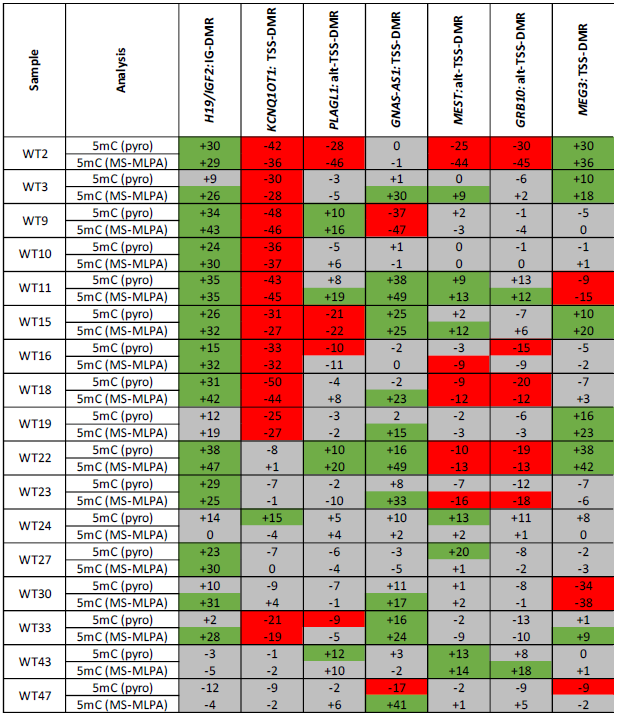


Boxes in green represent gain of methylation (GOM), in red loss of methylation (LOM), in grey of normal methylation (NM) values. The slight differences between pyroseq and MS-MLPA methylation values and the discrepancies found in three samples can be due to the analysis of different CpGs of the imprinted loci.

Table S4. DNA methylation and chromosome profile of WTs at indicated imprinted DMRs.


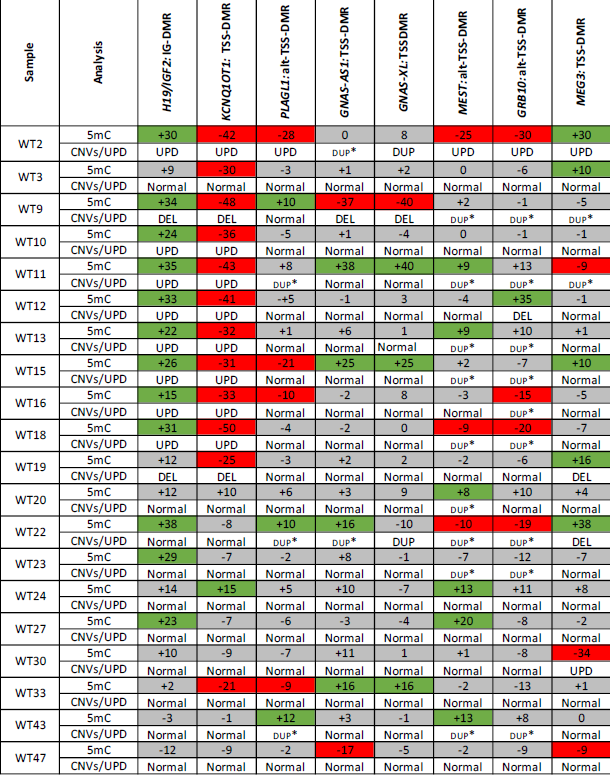


Boxes in green represent gain of methylation (GOM), in red loss of methylation (LOM), in grey of normal methylation (NM) values.

Table S5. Primers for pyrosequencing analysis.


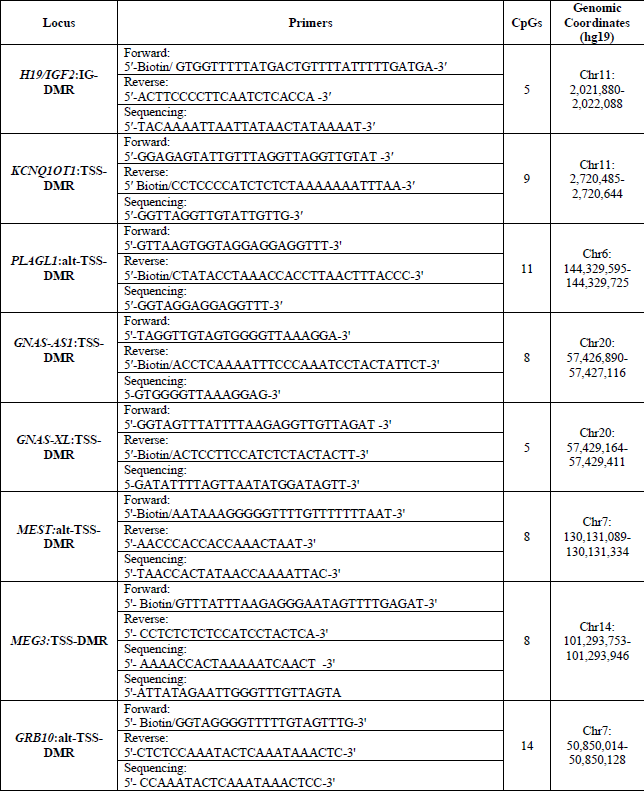

Supplement: Supplementary file 1 [file cancers-12-03411-s001.zip › cancers-995122-supplement.docx]
